# Exogenous copper exposure causing clinical wilson disease in a patient with copper deficiency

**DOI:** 10.1186/s12876-021-01859-6

**Published:** 2021-07-08

**Authors:** Blanca C. Lizaola-Mayo, Rolland C. Dickson, Dora M. Lam-Himlin, David M. Chascsa

**Affiliations:** 1 Division of Gastroenterology and Hepatology, Mayo Clinic, Phoenix, AZ USA; 2 Transplant Center, Mayo Clinic, Phoenix, AZ USA; 3 Division of Pathology, Mayo Clinic, Phoenix, AZ USA

**Keywords:** Human Swayback, Copper deficiency, Copper toxicity, Liver transplantation

## Abstract

**Background:**

Human Swayback is a disease characterized by acquired copper deficiency which primarily manifests as myeloneuropathy. Common causes include malabsorptive disorders, gastric surgery, total parenteral nutrition and excessive zinc intake. In contrast, copper supplementation should be closely monitored as excessive doses can lead to acute intoxication and in chronic cases, cirrhosis. Copper derangements are rare, however it is important to consider them due to potential severe complications.

**Case presentation:**

We present a middle-aged man who had been previously diagnosed with Human Swayback after presenting with various neurological symptoms. The patient was subsequently placed on copper supplementation. A decade later, he was referred to our hospital for liver transplant evaluation due to new diagnosis of decompensated end-stage liver disease after an abdominal surgery. His initial workup was suggestive of Wilson disease—subsequent ATP7B gene was negative. Ultimately, the patient underwent liver transplantation; liver explant was significant for a copper dry weight concentration of 5436 mcg/g.

**Conclusions:**

Human Swayback is a very rare copper-related disease which deserves awareness due to its potential irreversible health effects in the human body. Additionally, in patients who require copper supplementation, serial levels should be monitored to ensure adequate copper levels.

## Background

Copper is an essential trace mineral that acts as a cofactor for many enzymatic pathways in the human body. Its homeostasis is extremely delicate. There are two well-known inherited copper metabolic disorders—Menkes and Wilson disease. More recently, Human Swayback has been recognized as an acquired copper deficiency state in humans. Copper deficiency is known to cause myelopathy. In contrast, high copper concentrations can be cytotoxic causing a wide variety of symptoms including abdominal pain, liver failure and neurological disturbances. We present a rare case of Human Swayback disease and subsequent iatrogenic copper overload requiring liver transplantation. The aim of this case report is to provide an overview of copper deficiency, copper overload and its most common symptoms.

## Case Presentation

A 55 year-old man with past medical history of copper deficiency (Human Swayback), atrial fibrillation and new diagnosis of decompensated cryptogenic cirrhosis, was transferred to our facility for inpatient liver transplant evaluation due to acute on chronic liver failure with a MELD-Na score of 29.

Ten years prior to presentation, the patient started experiencing lightheadedness, dizziness, unsteady gait, mental fog, lower extremity weakness, memory loss, slurred speech, hand tremors, hallucinations and headaches. He underwent extensive neuropsychiatric and metabolic evaluation significant for a serum ceruloplasmin of 14.4 md/dL (normal 15–30 mg/dL), serum copper < 5 mcg/L (normal 15–60 mcg/L) with negative 24-h urine copper—diagnosed with Swayback disease. The patient was subsequently placed on copper supplementation 2 mg per day. The patient unfortunately was lost in follow up and he continued to take copper up to 8 mg per day. Three years after starting copper supplementation, the patient started experiencing severe diffuse abdominal pain, requiring multiple emergency department visits, opioid use, umbilical hernia repair and ultimately exploratory laparotomy. At the time of the exploratory laparotomy, the liver was noted to have cirrhotic morphology. After surgery, the patient developed signs of hepatic decompensation manifested by refractory ascites requiring paracentesis and hepatic encephalopathy. Due to worsening MELD-Na score and development of hepatorenal syndrome, the patient was transferred for inpatient liver transplant evaluation to our hospital.

**Fig. 1:**
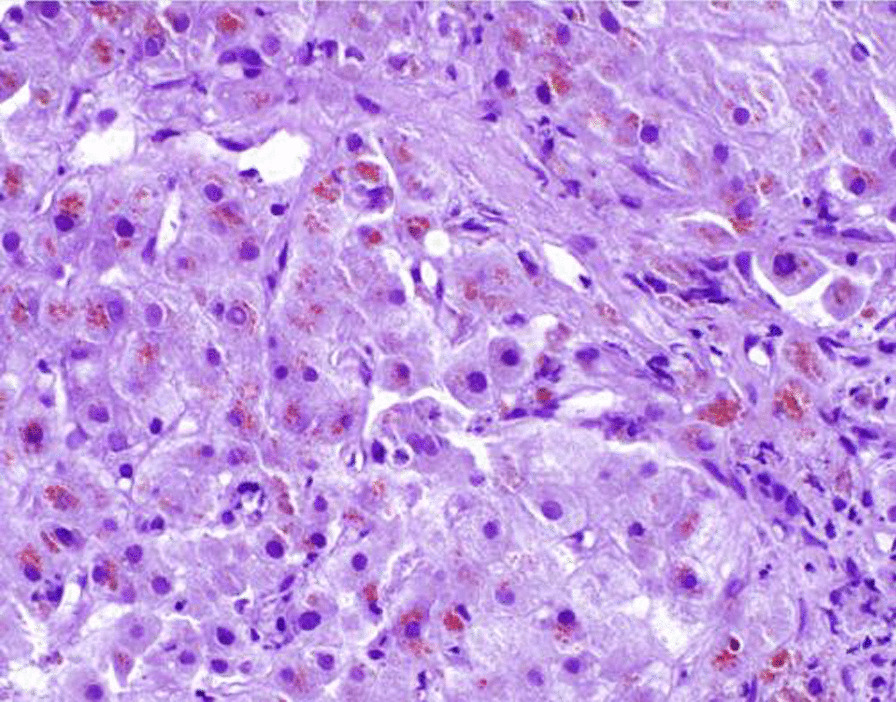
Widespread distinctive red-brown granules are found within hepatocytes as a result of copper accumulation (rhodamine copper stain, original magnification 400x)

**Fig. 2:**
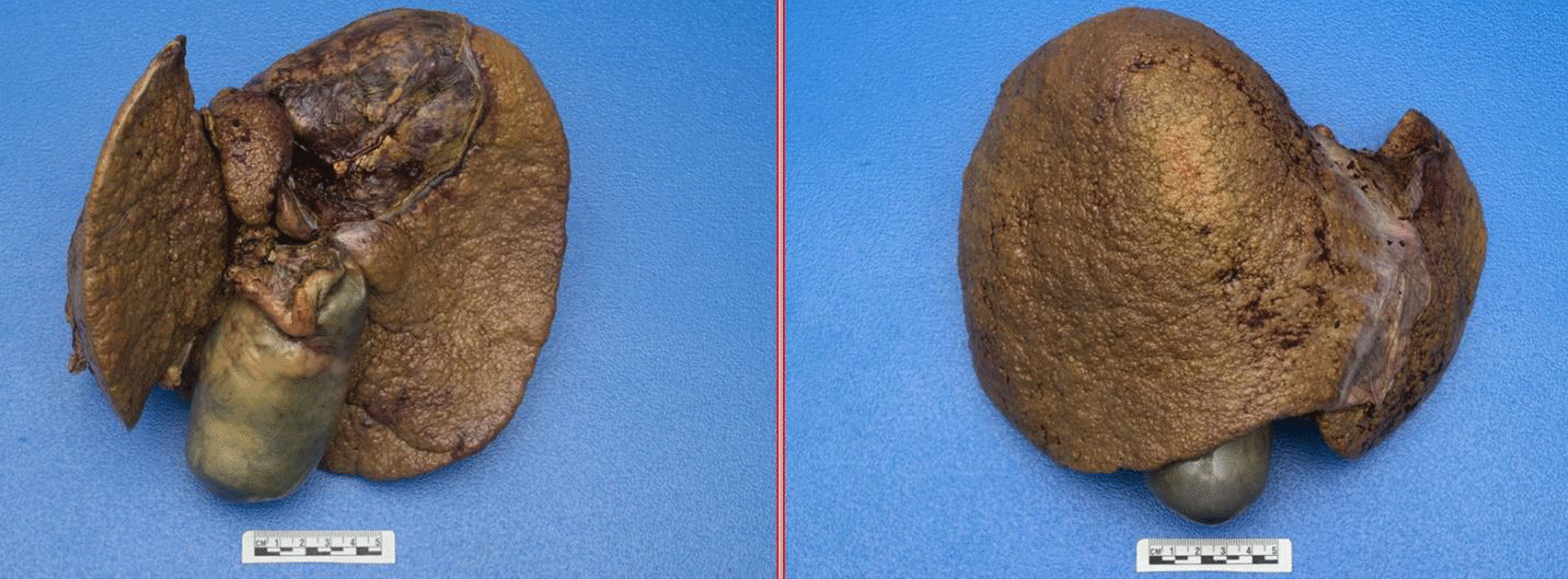
Liver explant. Pale golden-tan liver with micro-macronodules

Inpatient liver transplant evaluation was significant for hemolytic anemia hemoglobin 6.6 g/dL (baseline 13 g/dL), MCV 108.7 fL, low haptoglobin < 14 mg/dL and increased lactate dehydrogenase 239 U/L. Total bilirubin was 6.3 mg/dl, direct bilirubin 3.8 mg/dl, alanine aminotransferase 51 U/L, aspartate aminotransferase 190 U/L, alkaline phosphatase 68 U/L, creatinine 1.45 mg/dL, cystatin C 1.35 mg/L, ceruloplasmin 10.9 mg/dl, serum copper 1.73 mcg/ml and urine copper > 6210 mcg/24 h. Physical exam revealed scleral icterus, no Kayser-Fleischer rings on slit-lamp, abdominal distention with positive fluid shift and diffuse abdominal tenderness on palpation. Patient denied any alcohol use. A serological evaluation for other causes of liver disease was negative including the ATP7B gene for Wilson disease. A transjugular liver biopsy was performed that demonstrated ongoing portal/segmental inflammation with ballooning degeneration and diffuse hepatocellular copper accumulation on rhodamine stain (Fig. 1). Copper had been discontinued at admission. Zinc gluconate 50 mg by mouth three times daily was started. The patient was felt to be a good candidate for transplant, was listed with a MELD-Na score of 47 and ultimately underwent orthotopic liver transplantation. The liver explant was pale golden-tan with micro-macronodules. The copper concentration was 5436 mcg/g on dry weight (Fig. 2). The patient’s chronic abdominal pain subsided after liver transplant.

## Discussion and conclusions

Copper deficiency or Human Swayback is a rare condition recently recognized in humans [1–3]. This disease was originally described in ruminants as a cause of ataxic myelopathy. Some of the main causes of acquired copper deficiency in humans include hereditary disorders like Menkes disease, excessive zinc ingestion, large body surface burns, malabsorptive disorders, gastric surgery, chronic denture cream use, prolonged total parenteral nutrition and idiopathic [4–8].

The most common manifestations are anemia, leukopenia and myelopathy. Myelopathy may present with spastic gait, hyperreflexia, sensory ataxia, impaired vibratory proprioception, lower extremity paresthesias and foot drop [1, 9, 10]. The differential diagnosis of the myelopathy is broad, including vitamin B12 deficiency, nitrous oxide abuse, tabes dorsalis, amyotrophic lateral sclerosis, multiple sclerosis, Friedreich ataxia and intradural extramedullary tumors [11–14].

Diagnostic evaluation includes measurement of serum copper, ceruloplasmin, vitamin B12, iron studies, vitamin D, vitamin E, zinc, homocysteine and methylmalonic acid levels [15]. As an acute phase reactant, ceruloplasmin may be falsely elevated in underlying inflammatory conditions like liver disease, malignancy, pregnancy, infections and diabetes [16]. Spinal cord imaging may be needed as well [17]. A 24-h urine copper has a low sensitivity [2, 14].

Treatment regimens have not been well established and some case reports suggest an initial dose of 8 mg per day tapered over a month followed by 2 mg of elemental copper per day [7]. The daily recommended copper dose in adults is 1 to 3 mg per day [18, 19]. Its duration should be determined by periodic serum copper evaluation. In some circumstances, parenteral copper may be necessary. Unfortunately, despite therapy, patients may have residual neurological deficit. Hematological derangements tend to resolve completely after therapy [20].

In contrast, copper overload is rare. Copper overload The daily copper recommended dietary allowance (RDA) is 900 mcg in adults [21]. It is believed that copper injury is secondary to the interaction between the reduced form of copper and oxygen, leading to toxic oxygen species (TOS) production like superoxide anion, hydrogen peroxide and hydroxyl radicals which have a potential of being extremely toxic to the cells and tissue [22, 23]. Acute copper intoxication (1,000—10,000 mg) may manifest with metallic taste, nausea, vomiting, abdominal pain, heart failure, hepatic failure, renal failure, intravascular hemolysis and ultimately death [24, 25]. Chronic ingestion of copper above RDA can also lead to multi systemic involvement including neurological, ophthalmic, psychiatric, gastrointestinal manifestations as well as hemolytic anemia [26]. In particular, the liver may develop acute apoptosis and necrosis due to high TOS concentrations and low glutathione, which impairs the hepatocyte reducing capacity [27]. Chronically, copper overload will cause progressive liver injury which may progress to cirrhosis [28]. Our patient presented with abdominal pain, hemolytic anemia, psychiatric symptoms, hepatic and renal failure all likely due to the copper overload. At the time of presentation effective medical options were limited. Therapy is based on cessation of copper intake, supportive management, low-copper diet and copper chelating agents such as D-penicillamine and trientine in patients with genetic Wilson disease [29]. Zinc may be utilized as it interferes with the absorption of copper by induction of metallothionein in the enterocytes and the liver [30, 31]. Metallothionein has a high affinity for copper, inducing luminal copper binding, preventing its absorption. The copper is then excreted through feces [32]. Patients should avoid food with high copper concentration like chocolate, organ meats, nuts, shellfish and well water. Our patient already had cirrhosis with decompensated liver disease making chelation therapy of limited benefit, though we used zinc to try to prevent further copper absorption and transplant was the ultimate effective treatment.

We present a rare case of human Swayback disease with long-term supratherapeutic copper supplementation, ultimately requiring liver transplantation due to decompensated cirrhosis. This case report highlights the diverse symptomatology of copper derangements. Abdominal pain is a common symptom seen in Copper overload—our patient had complete resolution of his pain after liver transplantation. Copper disorders are rare and worth awareness as its complications are preventable. Prompt diagnosis, treatment and close monitoring of copper levels and supplementation are imperative.

## Data Availability

Data sharing not applicable to this article as no datasets were generated or analyzed during the current study.

## Abbreviations

RDA: Recommended dietary allowance
TOS: Toxic oxygen species
